# Effect of Trandolapril on Regression of Retinopathy in Hypertensive Patients with Type 2 Diabetes: A Prespecified Analysis of the Benedict Trial

**DOI:** 10.1155/2010/106384

**Published:** 2010-06-10

**Authors:** Piero Ruggenenti, Ilian Iliev, Marco Filipponi, Stefano Tadini, Annalisa Perna, Maria Ganeva, Bogdan Ene-Iordache, Paolo Cravedi, Roberto Trevisan, Antonio Bossi, Giuseppe Remuzzi

**Affiliations:** ^1^Clinical Research Center for Rare Diseases ‘Aldo & Cele Daccò', Mario Negri Institute for Pharmacological Research, Negri Bergamo Laboratories, Via Gavazzeni, 11, 24125 Bergamo, Italy; ^2^Unità di Nefrologia, Azienda Ospedaliera Ospedali Riuniti di Bergamo, 24128 Bergamo, Italy; ^3^Unità di Oftalmologia, Azienda Ospedaliera Ospedali Riuniti di Bergamo, 24128 Bergamo, Italy; ^4^Unità di Diabetologia, Azienda Ospedaliera Ospedali Riuniti di Bergamo, 24128 Bergamo, Italy; ^5^Unità di Diabetologia, Treviglio Hospital, 24047 Bergamo, Italy

## Abstract

*Background*. The effect of angiotensin converting enzyme inhibitors (ACEi) on regression of retinopathy in type 2 diabetics is still ill defined. *Methods*. We compared the incidence of retinopathy regression in 90 hypertensive type 2 diabetics randomized to at least 3-year blinded ACEi with trandolapril (2 mg/day) or non-ACEi therapy who had preproliferative or proliferative retinopathy at baseline. *Results*. Over a median (interquartile range) follow-up period of 35.8 (12.4–60.7) months, retinopathy regressed in 27 patients (30.0%). Regression occurred in 18 of 42 patients (42.9%) on ACEi and in 9 of 48 (18.8%) on non-ACEi therapy (adjusted for predefined baseline covariates HR (95% CI): 2.75 (1.18–6.42), *P* = .0193). Concomitant treatment with or without Non-Dihydropyridine Calcium Channel Blockers (ndCCBs) did not appreciably affect the incidence of retinopathy regression. *Conclusions*. Unlike ndCCB, ACEi therapy may have an additional effect to that of intensified BP and metabolic control in promoting regression of diabetic retinopathy.

## 1. Introduction

Despite the beneficial effects of photocoagulation, retinopathy remains the leading cause of blindness in people aged 30 to 69 years, that ultimately affects more than 60% of type 2 diabetics [1]. Duration of diabetes, poor metabolic control, and arterial hypertension have been associated with the development and progression of retinopathy, although their relative role appears to differ in different series and clinical conditions [1, 2]. Undoubtedly, strict metabolic control is essential for the prevention and treatment of retinopathy. Reducing Blood Pressure (BP), however, has been recognized as an additional, and probably even more effective, therapeutic intervention [2]. 

However, the specific effects on the retina of different medications used to control arterial hypertension in diabetic patients are still unclear. Studies suggest that inhibitors of the renin angiotensin system (RAS) may retard progression more effectively than other antihypertensive drugs. Still, their effect has been never formally compared with other agents such as non-dihydropyridine calcium channel blockers (ndCCBs). Moreover, no study primarily addressed whether regression of retinopathy can be achieved in those who already have retinal involvement. 

To formally explore these issues, we took advantage of a large cohort of hypertensive type 2 diabetics from the BErgamo Nephrologic Diabetes Complications Trial (BENEDICT) [3]. These patients were expected to have a high prevalence of retinopathy at study entry because of the concomitance of two strong and possibly synergistic risk factors, arterial hypertension and type 2 diabetes. They were randomized to receive at least 3 years of treatment with the ACEi trandolapril, the nondihydropyridine CCB (ndCCB) verapamil, their combination (VeraTran), or placebo plus other antihypertensive drugs titrated to a systolic/diastolic BP goal of 120/80 mmHg or less. Data showed that patients on ACEi therapy (either as trandolapril alone or the combination VeraTran) compared to those on non-ACEi therapy (verapamil or placebo) had a significantly lower incidence of persistent microalbuminuria, which is an early marker of diabetic nephropathy and a major risk factor for cardiovascular disease in this population. 

The primary aim of the present study was to evaluate whether, in type 2 diabetic patients, treatment with the ACEi trandolapril may promote regression of diabetic retinopathy more effectively than antihypertensive medications that do not directly interfere with angiotensin II production or activity, at comparable BP and metabolic control. Secondarily, we compared the effects of trandolapril and non-RAS inhibitor therapy on newly onset retinopathy in those patients without evidence of retinal involvement at study entry. The results of the analyses formed the basis of the present report.

## 2. Methods

### 2.1. Patients and Study Design

This study is a pre-specified analysis of data from the BENEDICT trial. Study design and patient characteristics have been described in detail elsewhere [3]. Briefly, BENEDICT was a prospective, randomized, double blind, parallel group study that evaluated the possibility of preventing the onset of persistent microalbuminuria in 1209 patients with type 2 diabetes (WHO criteria), arterial hypertension (systolic or diastolic BP more than 130 or 85 mmHg, or concomitant antihypertensive therapy), and normal Urinary Albumin Excretion (UAE) rate (UAE < 20 *μ*g/min in at least 2 of 3 consecutive overnight urine collections) randomly assigned to at least 3 years of treatment with one of the following study drugs: I, a ndCCB: verapamil SR, 240 mg/day; II, an ACEi: trandolapril 2 mg/day; III, the fixed-dose combination of verapamil SR, 180 mg/day plus trandolapril 2 mg/day: VeraTran; and IV, placebo. The target BP after randomization and throughout the whole study period was to be less than 120/80 mmHg for all the treatment groups. Other antihypertensive drugs (with the exception of RAS inhibitors and ndCCBs different from the study drugs) could be used to achieve and maintain target BP according to predefined guidelines. 

**Figure 1:**
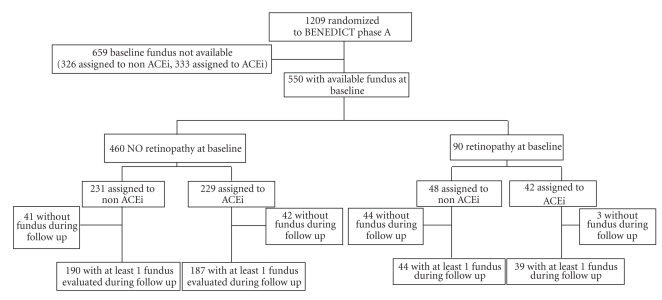
Study flow chart.

The analysis was primarily aimed at evaluating the rate of regression of diabetic retinopathy in patients with retinal involvement at study entry considered as a whole and, then, according to their original randomization to RAS-inhibitor or non-RAS inhibitor therapy (Figure 1). Secondarily, the study compared the effect of RAS and non-RAS inhibitor therapy on newly onset retinopathy in those without evidence of retinal involvement at inclusion. Finally, for explorative purposes only, patients were considered according to their randomization to one of the four original treatment arms.

The study protocol was in accordance with the declaration of Helsinki and was approved by the institutional review board at each Center and by the Safety Committee of the BENEDICT study. All patients gave written informed consent.

### 2.2. Retinal Evaluation

Retinal evaluations by ophthalmoscopy and photography (in a subgroup) were scheduled at baseline, every year thereafter, and at final visit in all patients included in the BENEDICT trial who had been randomized at the Clinical Research Center (CRC) “Aldo and Cele Daccò” of The Mario Negri Institute and at the Unit of Diabetology of the Azienda Ospedaliera “Ospedali Riuniti di Bergamo”. They were referred to the Unit of Ophthalmology of the Azienda Ospedaliera where they were evaluated independently by two ophthalmologists (I. I. AND M. F.) blinded to the clinical and laboratory data of the patients. The diagnoses were compared for consistency. Patients with an inconsistent diagnosis were evaluated by a third independent ophthalmologist (S.T.), and his diagnosis was recorded as final and considered for data analyses [5]. After mydriasis was induced, indirect binocular ophthalmoscopy was performed by a L-0185 slit-lamp biomicroscope (magnification 10x and 16x) and handheld lens (magnification 90x). Photographs of four standard 30° fields of each eye were taken through dilated pupils in stereo pairs (lateral to macula, macula, disc, and nasal) with Canon CF 60 UV fundus camera (Tokio, Japan) [4]. The pictures were printed on Kodak Ektachrome 100-colour slide film. Photographs were initially assessed for quality and adherence to the protocol. Inadequate photographs were discharged. 

The eye with the most severe involvement was used for categorization of retinal involvement. Pre-proliferative retinopathy was defined by the presence of microaneurysms, hemorrhages, hard exudates, venous congestion, cotton wool spots, or intraretinal microvascular abnormalities. Proliferative retinopathy was diagnosed when new vessels, glial proliferation, preretinal hemorrhage, vitreous hemorrhage, scars of photocoagulation (known to have been directed at new vessels), and/or retinal detachment were found. Patients with none of these abnormalities were classified as not having retinopathy [5, 6]. Based on this simplified classification, regression of retinopathy was defined as a persistent (up to the final visit) change in the stage of retinal involvement from proliferative to pre-proliferative retinopathy, or from pre-proliferative retinopathy to no retinal involvement.

### 2.3. BP and Other Outcome Variables

Trough systolic and diastolic (Korotkoff phase I/V) BPs were measured in the morning before treatment administration by use of an appropriate cuff with a sphygmomanometer and with the patient in a sitting position after at least 5 minutes rest. Three measurements to the nearest 2 mmHg were obtained, two minutes apart at each time point, and the average of the three measurements was recorded for statistical analyses. Mean arterial pressure (MAP) was calculated as diastolic BP plus one third of the pulse pressure. All the laboratory measurements were centralized at the Laboratory of the CRC. HbA1C was measured by ion exchange high-performance liquid chromatography and urinary albumin excretion rate by nephelometry. 

Data were reported in dedicated case record forms and doubly entered in an ad hoc database that was eventually merged with the BENEDICT database. Before analyses, all data were monitored by the Monitoring Unit of the CRC.

### 2.4. Sample Size

Regression of retinopathy was the primary outcome variable of a substudy ancillary to BENEDICT phase A. At the time the present analyses were planned, no data were available on the regression of retinopathy in hypertensive patients with type 2 diabetes on intensified BP and metabolic control, as well as on a possible additional effect on disease regression of ACEi therapy. Thus, it was impossible to establish *a priori* the sample size required to provide the analyses with an adequate power to detect the hypothesized treatment effect on retinopathy. Actually, this was an explorative study performed in all consenting patients with available ophthalmologic evaluations.

### 2.5. Statistical Analyses

The analyses were performed by the Laboratory of Biostatistics of the CRC. Patients were eligible if they had a funduscopy evaluation at baseline. Main outcome variable was regression of retinal changes in patients with retinopathy at study entry. Secondary outcome variable was newly onset retinopathy in those with no retinal changes at study entry. In the primary outcome analyses, patients with retinopathy were considered as a whole regardless of the stage of retinal involvement. For outcome analyses, systolic and diastolic BP measurements were included separately in the model. Continuous variables were compared by unpaired *t*-test or Wilcoxon Rank Sum tests and categorical variables by *χ* ^2^ test or Fisher's Exact test. Regression of retinopathy was evaluated by means of Cox regression models in order to obtain the hazard ratio (HR) and its 95 percent confidence interval. Patients without funduscopy evaluation on follow up were conventionally classified as having one day of follow up and without the event of interest. Unless otherwise stated, statistical analyses were done according to the intention-to-treat principle and considered adjustments according to pre-defined baseline covariates (site, age, smoking status, diastolic BP, and log-transformed urinary albumin excretion). All the statistical analyses were performed using SAS version 9 (SAS Institute Inc, Cary, NC). A *P*-value of less than  .05 was considered as statistically significant. No *P*-value adjustment was carried out for multiple comparisons. Data are expressed as mean ± standard deviation (SD) or median and interquartile (IQ) range or percentages.

## 3. Results

### 3.1. Baseline Characteristics

Of the 1209 patients randomized in the original BENEDICT cohort, 583 patients were referred to the two centers involved in the present study. Five-hundred-fifty patients had a baseline funduscopy evaluation (Figure 1). Patients with funduscopy evaluation, compared to those without, had a lower body mass index, poorer metabolic control and higher BP at baseline (Table 1). Four-hundred-sixty patients (83.6%) had no evidence of retinal involvement. Of the remaining 90 patients with funduscopy data, 82 had a pre-proliferative and 8 had a proliferative form of retinopathy. All had BP and HbA1C data at baseline and on follow-up and were therefore available for this analysis. Compared to patients without evidence of retinal involvement, those with retinopathy (either pre-proliferative or proliferative) at inclusion reported a significantly longer duration of diabetes, were more hypertensive and had significantly higher HbA1C, blood glucose levels, and urinary albumin excretion (Table 1). Age, gender distribution, smoking habit, serum creatinine, and lipid profile were similar between groups. A similar proportion of patients with retinopathy were on ACEL or non-ACEi therapy, and a similar proportion of patients were on ndCCB or non-ndCCB therapy (Table 1). Baseline characteristics of patients on ACEL or non-ACEi therapy, as well as of patients on ndCCB or non-ndCCB therapy were comparable, with the only exception of systolic BP that was lower in those on ndCCB compared to those on non-ndCCB therapy (Table 1). The proportion of patients on concomitant medications at baseline and on follow-up was also similar within each considered treatment group, with the only exception of the proportion of patients on fibrate therapy at baseline that was lower in the ndCCB than in the non-ndCCB treatment group (Table 2).

### 3.2. Regression of Diabetic Retinopathy According to ACEi, or Non-ACEi Therapy

**Figure 2:**
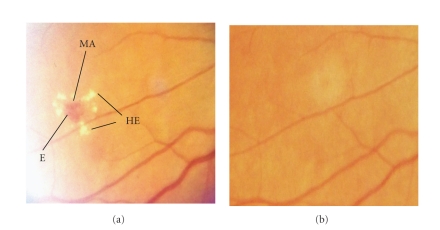
Fundus photographs showing pre-proliferative changes (a) at baseline in a patient who had a regression of eye lesions after three years of trandolapril therapy (b). This picture provides a comprehensive example of three typical lesions, microaneurysms (MA), hemorrages (E), and hard exudates (HE, that may regress in type 2 diabetic patients on ACE inhibitor therapy combined to intensified metabolic and blood pressure control, as in the BENEDICT trial.

**Figure 3:**
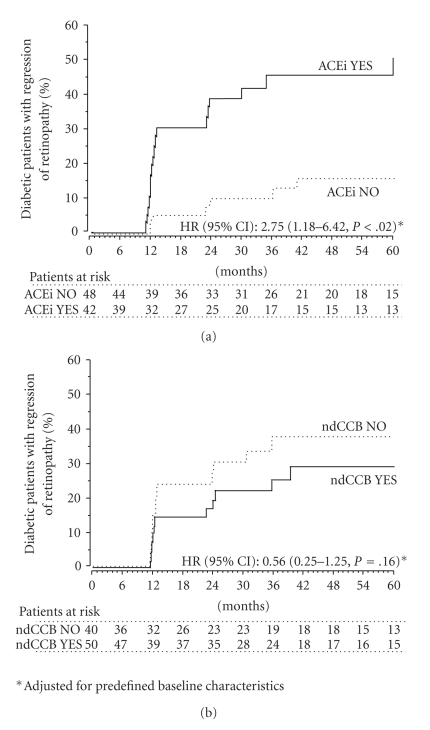
Cumulative incidence of patients with retinal involvement at baseline who achieved regression of diabetic retinopathy according to randomization to ACEi therapy YES or NO (a) or to ndCCB therapy YES or NO (b).

Over a median (IQ range) follow-up period of 35.8 (12.4–60.7) months, retinal changes regressed in 27 of 90 patients (30.0%) who had retinopathy at study entry. Regression was observed in 18 of the 42 patients (42.9%) randomized to ACEi therapy and in 9 of the 48 patients (18.8%) randomized to non-ACEi therapy (Figure 2) (HR (95% CI): 2.62 (1.17–5.84), *P* = .0188, (unadjusted) and 2.75 (1.18–6.42), *P* = .0193 (adjusted for predefined baseline covariates)) (Figure 3(a)). Systolic and diastolic BP were similar in the two treatment groups at baseline (Table 1) and at different visits on follow-up. HbA1C was also similar between groups at baseline (Table 1) and on follow up. The regression rate of retinopathy was significantly different even after adjustment for baseline and follow-up systolic/diastolic BP and HbA1C and for systolic/diastolic BP and HbA1C changes versus baseline (*P* < .05 for all considered adjusted Hazard Ratios).

### 3.3. Regression of Diabetic Retinopathy According to ndCCB or Non-ndCCB Therapy

Regression of retinopathy was observed in 12 of the 50 patients (24.0%) randomized to ndCCB therapy and in 15 of the 40 patients (37.5%) randomized to non ndCCB therapy (HR (95% CI): 0.64 (0.30 to 1.37), *P* = .25 (unadjusted) and 0.56 (0.25 to 1.25), *P* = .16 (adjusted for predefined baseline covariates)) (Figure 3(b)). Systolic BP was lower in the ndCCB than in the non-ndCCB group at baseline (Table 1), but the difference progressively weaned on subsequent follow up visits, while diastolic BP was similar in the two treatment groups at baseline (Table 1) as well as at different visits on follow-up. HbA1C was similar between groups at baseline (Table 1) and at different visits on follow up.

### 3.4. Regression of Diabetic Retinopathy According to the Original Treatment Arm

Regression of retinopathy was observed in 10 (52.6%), 8 (34.8%), 2 (7.4%), and 5 (23.8%) of the 19, 23, 27, and 21 patients randomized to trandolapril, VeraTran, verapamil, or placebo, respectively. The HR (95% CI) for trandolapril, VeraTran, or verapamil versus placebo was, respectively: 2.47 (0.84–7.23), *P* = .10; 1.72 (0.55–5.32), *P* = .35; 0.61 (0.16–2.27), *P* = .46 (unadjusted) and: 2.61 (0.84–8.13), *P* = .10; 1.89 (0.53–6.71), *P* = .33; and  0.91 (0.19–4.33), *P* = .90 (adjusted for predefined baseline covariates.) Systolic and diastolic BP and HbA1C were not significantly different between treatment groups both at baseline (Table 1) and on follow-up (data not shown).

### 3.5. Newly Onset Diabetic Retinopathy

Retinal changes developed in 61 of 460 patients (13.3%) who had no evidence of diabetic retinopathy at study entry. Newly onset retinopathy was observed in 33 of the 229 patients (14.4%) randomized to RAS inhibitor therapy and in 28 of the 231 patients (12.1%) randomized to non-RAS inhibitor therapy (unadjusted: HR (95% CI) 0.968 (0.582–1.610), *P* = .90; adjusted for predefined baseline covariates: HR (95% CI) 0.984 (0.588–1.646), *P* = .95). No significant difference between groups was detected even after adjustment for pre-defined baseline and follow up covariates, including baseline and follow up BP and HbA1 C (data not shown). No difference in new onset retinopathy was observed between patients on ndCCB and non-ndCCB therapy (data not shown).

## 4. Discussion

Our study shows that regression of diabetic retinopathy is possible in a substantial proportion of hypertensive patients with type 2 diabetes and tight BP and metabolic control. Importantly, we found that therapy with antihypertensive drugs that directly interfere with the RAS, such as the ACEi trandolapril, is more effective in inducing regression than non-RAS inhibiting therapy, while treatment regimens including or not including the ndCCB verapamil have similar effects. Secondarily, data showed that the protective effect of trandolapril against diabetic retinopathy is not appreciably enhanced by combined therapy with verapamil, and the effect of verapamil is not different from that of placebo. On the other hand, trandolapril as well as verapamil had no specific protective effect against the development of retinopathy in patients with no evidence of retinal involvement at study entry.

These findings are in line with the recent DIRECT-Protect 2 trial showing that the angiotensin receptor blocker (ARB) candesartan increases regression of retinopathy by 34% over placebo in type 2 diabetic patients with mild to moderately severe retinal lesions but has no appreciable effect on progression of retinopathy in those patients without retinal involvement at inclusion [7].

Hypertension may increase the shear stress on the vascular wall, leading to hyperplasia of the vascular endothelium and to hypertrophia of its cytoskeleton [8]. This process may be magnified in the diabetic retina, since defective autoregulation may favor the transmission of high systemic BP down to the microcirculation, causing capillary hypertension and structural damage to the endothelium [9]. Thus, lowering BP might decrease the barotrauma to the vascular wall and therefore prevent or regress the microvascular changes of the diabetic retina.

Throughout the observation period the regression rate of diabetic retinopathy was more than double in patients on trandolapril than in controls. This finding was not explained by blood pressure control that was similar across different treatment groups and was consistent with a specific beneficial effect of trandolapril on the retina.

Activation of the RAS has been involved in defective autoregulation of the retinal microvasculature, and inhibition of the RAS may therefore explain the improved perfusion observed during ACE inhibition therapy. Moreover, the hemodynamic effects of RAS inhibition have been suggested to contribute to the partial regression of retinal changes observed during ACEi therapy in hypertensive rats with streptozotocin induced diabetes [10]. 

ACE inhibitors, however, may also interfere with a series of nonhemodynamic effects mediated by the RAS. The RAS is involved in growth factor expression, in particular of vascular endothelial growth factor (VEGF) and tumor growth factor (TGF)-beta. Increased VEGF expression in the diabetic rat is normalized by ACE inhibition, which eventually translates into an amelioration of retinal injury. In the presence of high glucose, angiotensin II stimulates TGF-beta secretion, which increases matrix accumulation by activating the synthesis of collagen I and fibronectin and by decreasing matrix degradation [11]. Consistently, blockade of angiotensin II synthesis by ACEi decreases the expression of TGF-beta, reduces the accumulation of matrix protein synthesis, and accelerates its degradation.

Finally, ACEi may interfere with RAS-independent metabolic pathways. By inhibiting bradykinin degradation, they may increase the bioavailability of nitric oxide and prostacyclin which, through an increased bioavailability and activity of Na^+^, K^+^-ATPase in the vasculature of the diabetic retina, might contribute to the functional improvement detected by electroretinography [12] during ACEi therapy. 

Finding that trandolapril was not protective against the development of retinopathy confirms and extends data from DIRECT-Protect 2 trial that the angiotensin II receptor blocker candesartan had no appreciable effect on progression of retinopathy in type 2 diabetic patients without retinal involvement at inclusion [7]. A possible explanation is that all patients were on intensified BP and metabolic control, which substantially decreased the overall incidence of events, reducing the statistical power of comparative analyses between treatment groups. Indeed, while optimized BP and metabolic control increased the number of regressions in those with retinal involvement at baseline, which increased the power of comparative analyses between treatment groups, in those without retinal disease optimized treatment decreased the incidence of newly onset retinopathy, which decreased the statistical power of between-group comparisons. An alternative or complementary explanation would be that mechanisms sustaining progression of retinopathy may differ from those at the basis of disease regression, which might translate into different response to ACEi therapy of patients with or without retinal changes to start with.

On the other hand, present data on the retina, combined with the results of the BENEDICT Phase 1 study showing that, in patients with type 2 diabetes and normal urinary albumin excretion, verapamil therapy failed to prevent microalbuminuria [3], confirm that this drug has no specific protective effects against microvascular disease of type 2 diabetes. 

### 4.1. Limitations

We graded the severity of retinal involvement by a simplified score that has been validated in previous studies by our [5] and other [6, 13–15] groups and has been recently used in other large-scale, prospective, and randomized clinical trials [16]. Compared to a more complex score implemented to grade retinal involvement in people with diabetes [17], this approach discriminates only two stages (pre-proliferative and proliferative) of retinopathy. Thus changes in one or more grades within the same pre-proliferative or proliferative stage could not be captured by this approach. This reduced the sensitivity and precision of the assessment and, secondarily, the power of the analyses but did not introduce a systematic bias since the same limitation was applied to the same extent to each considered patient group. On the other hand, compared to more complex approaches, the criteria we used to grade retinal involvement in our present study more closely reflect the criteria normally used in every-day clinical practice, which enhances the generalizability of our present findings to the average population of patients with type 2 diabetes. 

Regression of retinopathy was the main outcome variable of a substudy ancillary to BENEDICT phase A. Thus, the substudy was not powered *a priori* on the basis of an expected treatment effect on the regression of retinal involvement but rather aimed at including all BENEDICT patients with available funduscopy evaluation at baseline. However, a posteriori evaluations showed that, due to the strong treatment effect of trandolapril, the probability of a false positive finding was less than two percent. 

Finally, analysis was restricted to patients referred to two of the nine Centers involved in the BENEDICT trial. This was because of the logistic possibility for these Centers to refer randomized patients to the Unit of Ophthalmology where funduscopy evaluations were performed (these three Institutions were in the same urban area). However, since randomization to different treatment arms was balanced within each Center, this did not introduce any appreciable bias. This is consistent with the evidence that patient distribution was balanced, and baseline characteristics were similar among different treatment groups.

## 5. Conclusions

The present study, along with the recently published DIRECT-Protect 2 trial, provided the evidence that diabetic retinopathy can regress. This may have important clinical implications since regression of retinal damage may limit the risk of visual loss in the long term. Moreover, microvascular complications of diabetes, such as nephropathy and retinopathy, may reflect coronary ischemic disease and predict cardiovascular morbidity and mortality [18]. Conceivably, as already reported for regression of renal disease, regression of retinopathy might also predict reduced cardiovascular risk in the long-term. 

Our present data showed that RAS inhibition with the ACEi trandolapril, unlike calcium channel blockade by verapamil, had a beneficial effect that exceeded the benefit expected from the reduction in arterial BP and blood glucose observed during the study. Importantly, the ACEi trandolapril, compared to the ARB candesartan tested in the DIRECT-Protect 2 trial, has the advantage of remarkably lower treatment costs (US$ 1.10 versus 2.60, $ 0.24 versus 0.58, or Euro 0.49 versus 0.94 for one day therapy with trandolapril 2 mg or candesartan 32 mg, resp.). Costs can be further reduced by using the generic compound that is currently available in most countries. Improving cost-effectiveness of intervention programs at population level would have major implications, as diabetic retinopathy is a leading cause of visual impairment worldwide [1], and its incidence is expected to further increase along with the forecasted epidemic of diabetes, in particular in developing countries [19]. 

Considering the tremendous burden of diabetes and of its chronic complications, these findings may have important clinical and social implications for patients, physicians, and other health care providers.

## Figures and Tables

**Table 1:** Baseline characteristics of hypertensive patients with type 2 diabetes and normoalbuminuria according to availability of fundus evaluation YES/NO, evidence of retinopathy at study entry YES/NO, and to randomization to ACE inhibitor therapy YES/NO and ndCCB therapy YES/NO.

	Funduscopy Yes	Funduscopy No	Retinopathy Yes	Retinopathy No	With Retinopathy			
					ACEi Yes	ACEi No	ndCCB Yes	ndCCB No

Number of patients	550	659	90	460	42	48	50	40
*Demography*								
Age (yrs)	62.0 ± 7.8	62.6 ± 8.2	61.3 ± 7.9	62.1 ± 7.8	61.5 ± 7.7	61.1 ± 8.1	60.8 ± 8.4	62.0 ± 7.1
Males (*n*)	294 (53.5)	345 (52.3)	48 (53.3)	246 (53.5)	24 (57.1)	24 (50.0)	30 (60.0)	18 (45.0)
Clinics								
BMI (kg/m^2^)	28.7 ± 4.5	29.4 ± 4.9*	28.3 ± 4.0	28.8 ± 4.6	28.0 ± 3.3	28.6 ± 4.5	28.4 ± 4.0	28.1 ± 4.0
Diabetes duration (yrs)	7.9 ± 6.6	7.4 ± 6.5	10.5 ± 7.2	7.4 ± 6.3°°°	11.2 ± 7.5	9.9 ± 7.1	10.9 ± 7.0	10.1 ± 7.6
Smokers								
Never	323 (58.7)	377 (57.2)	58 (64.4)	265 (57.6)	24 (57.1)	34 (70.8)	31 (62.0)	27 (67.5)
Former	169 (30.7)	194 (29.4)	24 (26.7)	145 (31.5)	14 (33.3)	10 (20.8)	15 (30.0)	9 (22.5)
Current	58 (10.5)	88 (13.3)	8 (8.9)	50 (10.9)	4 (9.5)	4 (8.3)	4 (8.0)	4 (10.0)

*Laboratory *								
HbA1c (%)	5.9 ± 1.5	5.7 ± 1.3**	6.5 ± 1.5	5.8 ± 1.4°°°	6.6 ± 1.4	6.5 ± 1.7	6.6 ± 1.7	6.4 ± 1.2
Systolic BP (mm Hg)	151.6 ± 14.3	149.3 ± 12.9*	158.4±16.5	150.3 ± 13.5°°°	161.2 ± 15.3	155.9±17.2	154.3±15.2	189.0 ± 55.6
Diastolic BP (mm Hg)	88.8 ± 8.3	86.4 ± 6.9***	90.7 ± 8.2	88.4 ± 8.3°	92.4 ± 7.9	89.1±8.3	89.9±8.5	91.6 ± 7.9
Albuminuria (*μ*g/min)	6.8 ± 4.5	7.0 ± 4.6	8.1 ± 5.1	6.6 ± 4.4°°	8.0 ± 5.0	8.2 ± 5.2	8.3 ± 5.4	7.8 ± 4.6
Ser. creatinine (mg/dL)	0.9 ± 0.2	0.9 ± 0.2	0.9 ± 0.2	0.9 ± 0.2	0.9 ± 0.2	0.9 ± 0.1	0.9 ± 0.1	0.9 ± 0.2
Triglycerides (mg/dL)	143.5 ± 73.2	151.7 ± 83.4	146.3 ± 75.0	142.9 ± 72.9	146.7 ± 64.9	145.9 ± 83.6	139.7 ± 80.1	154.4 ± 68.2
Tot. Cholesterol (mg/dL)	212.0 ± 36.5	208.5 ± 35.1	213.1 ± 40.5	211.8 ± 35.7	207.7 ± 31.2	217.8 ± 46.9	209.4 ± 37.3	217.7 ± 44.1

Data are mean ± SD or numbers and percentages (in brackets).

*P < .05, **P < .01, ***P ≤ .001 versus Fundoscopy YES; °*P* < .05, °°*P* < .01, °°°*P* ≤ .001 versus Retinopathy YES; ^^^ *P* < .01 versus ndCCB YES.

**Table 2:** Concomitant medications in patients with type 2 diabetes and microalbuminuria at baseline and during follow-up according to treatment with ACE inhibitors YES or NO or with ndCCB YES or NO.

	Baseline				Follow-up			
	ACEi Yes	ACEi No	ndCCB Yes	ndCCB No	ACEi Yes	ACEi No	ndCCB Yes	ndCCB No
Number of patients	42	48	50	40	39	44	47	36

Concomitant medication	number (percent)				number (percent)			
*Glucose-lowering regimen*								
Diet alone	5 (11.9)	12 (25.0)	12 (24.0)	5 (12.5)	4 (10.3)	8 (18.2)	8 (17.0)	4 (11.1)
Oral hypoglycemic agent alone	29 (69.0)	21 (43.8)	26 (52.0)	24 (60.0)	24 (61.5)	21 (47.7)	26 (55.3)	19 (52.8)
Insulin and oral hypoglycemic agent	5 (11.9)	12 (25.0)	10 (20.0)	7 (17.5)	11 (28.2)	16 (36.4)	13 (27.7)	14 (38.9)
Insulin alone	3 (7.1)	3 (6.3)	2 (4.0)	4 (10.0)	3 (7.7)	3 (6.8)	3 (6.4)	3 (8.3)

*Antihypertensive agents*								
Any	22 (52.4)	26 (54.2)	23 (46.0)	25 (62.5)	32 (82.1)	38 (86.4)	37 (78.7)	33 (91.7)
Diuretic	5 (11.9)	11 (22.9)	9 (18.0)	7 (17.5)	10 (25.6)	14 (31.8)	16 (34.0)	8 (22.2)
Beta-blocker	6 (14.3)	2 (4.2)	3 (6.0)	5 (12.5)	4 (10.3)	3 (6.8)	4 (8.5)	3 (8.3)
Calcium-channel blocker (dihydropyridine)	11 (26.2)	15 (31.3)	12 (24.0)	14 (35.0)	14 (35.9)	16 (36.4)	14 (29.8)	16 (44.4)
Sympatholytic agent	7 (16.7)	8 (16.7)	9 (18.0)	6 (15.0)	28 (71.8)	32 (72.7)	29 (61.7)	31 (86.1)

*Lipid-lowering agents*								
Any	3 (7.1)	3 (6.3)	1 (2.0)	5 (12.5)	6 (15.4)	9 (20.5)	6 (12.8)	9 (25.0)
Statin alone	0	1 (2.1)	0	1 (2.5)	2 (5.1)	7 (15.9)	4 (8.5)	5 (13.9)
Fibrate alone	3 (7.1)	1 (2.1)	0	4 (10.0)*	2 (5.1)	0	1 (2.1)	1 (2.8)
Statin and fibrate	0	0	0	0	2 (5.1)	1 (2.3)	0	3 (8.3)

*Antiplatelet agent*	1 (2.4)	0	1 (2.0)	0	6 (15.4)	3 (6.8)	6 (12.8)	3 (8.3)

*P < .05 versus ndCCB YES.
